# Remodeling Cildb, a popular database for cilia and links for ciliopathies

**DOI:** 10.1186/2046-2530-3-9

**Published:** 2014-11-17

**Authors:** Olivier Arnaiz, Jean Cohen, Anne-Marie Tassin, France Koll

**Affiliations:** 1 Centre de Génétique Moléculaire, CNRS, Avenue de la Terrasse, Gif sur Yvette, 91198, France

## Abstract

**Background:**

New generation technologies in cell and molecular biology generate large amounts of data hard to exploit for individual proteins. This is particularly true for ciliary and centrosomal research. Cildb is a multi–species knowledgebase gathering high throughput studies, which allows advanced searches to identify proteins involved in centrosome, basal body or cilia biogenesis, composition and function. Combined to localization of genetic diseases on human chromosomes given by OMIM links, candidate ciliopathy proteins can be compiled through Cildb searches.

**Methods:**

Othology between recent versions of the whole proteomes was computed using Inparanoid and ciliary high throughput studies were remapped on these recent versions.

**Results:**

Due to constant evolution of the ciliary and centrosomal field, Cildb has been recently upgraded twice, with new species whole proteomes and new ciliary studies, and the latter version displays a novel BioMart interface, much more intuitive than the previous ones.

**Conclusions:**

This already popular database is designed now for easier use and is up to date in regard to high throughput ciliary studies.

## Background

Whatever the field studied in biology, due to the prevalence of new generation technologies, retrieving relevant information from high throughput studies represents a most important challenge. In this view, five years ago, we developed Cildb, a knowledgebase that allowed data mining concerning cilia and ciliopathies (http://cildb.cgm.cnrs-gif.fr/) [1]. Cildb progressively became a reference cilium database, with a number of users reaching now 700 per month. Since its creation and publication [1], Cildb underwent several modifications and improvements, yielding an evolution to Version 2.1 in 2010 and now to Version 3.0 in 2014. Although data in Cildb are raw data treated automatically, so that false positives and false negatives may occur, results are fully informative and make easier searches on ciliary genes.

The purpose of this note is fourfold, reminding the reader of the main uses of this database already described in more detail by Arnaiz et al. [1], providing explanation of the updates, describing the new interface and evaluating the orthology relationships as calculated in Cildb.

### Cildb, a database for ciliary studies… and more

In the early 2000’s, high throughput studies started to appear concerning cilia, a re-emerging organelle at that time [2], and centrioles [3], precursors of basal bodies of cilia in metazoans. Such studies generated large amounts of data on cilia, basal body, centriole, and centrosome proteomes, on transcriptome analyses realized under various conditions (ciliogenesis etc.), and on computation issued from comparative genomics between centric (i.e. with cilia/flagella or at least centrioles at some stage of their life cycle) and acentric organisms. Developing a way to browse these data became essential, not only from the statistician’s point of view, but also for experimental biologists who want to seek information on individual proteins from the bulk of the results.

## Methods

The originality of Cildb was in its backbone that related on the one side a network of orthology between the whole proteomes, complete sets of protein sequences, of all the species taken pair-wise, calculated with the algorithm of Inparanoid version 4.1 with default parameters [4], and on the other side the detection of each protein in a set of ciliary studies [1]. Therefore, the database allows searches for possible ciliary properties on the whole proteome of one species, e.g. *Homo sapiens*, based on ciliary properties established by studies conducted in another species, e.g. flagellum proteomics in *Chlamydomonas*[5]. In addition, the whole human proteome has been linked to the OMIM database (http://www.ncbi.nlm.nih.gov/omim/) that gathers all known human genetic disorders with the corresponding genes. This allows searches of proteins involved in diseases and to display the OMIM description as attribute in the output of a search. Conversely, searches in the whole proteome of any non-human species can tell if the resultant proteins are orthologous to human proteins linked to human diseases.

In addition to the ciliary properties of proteins, Cildb contains other information such as synonyms, descriptions, molecular weight, isoelectric point, probability of presence of a signal peptide, of transmembrane helices, as well as the FASTA sequence. This extra information can be searched for and displayed as properties using Cildb.

Cildb has been imagined and worked out to manipulate outputs of high throughput studies. All data coming from studies dedicated to the function of only a specific or of several proteins are not included in Cildb so that some ciliary proteins may escape from Cildb searches if they are not revealed by high throughput studies.

## Results and discussion

### What is new in Cildb V3.0?

**Figure 1:**
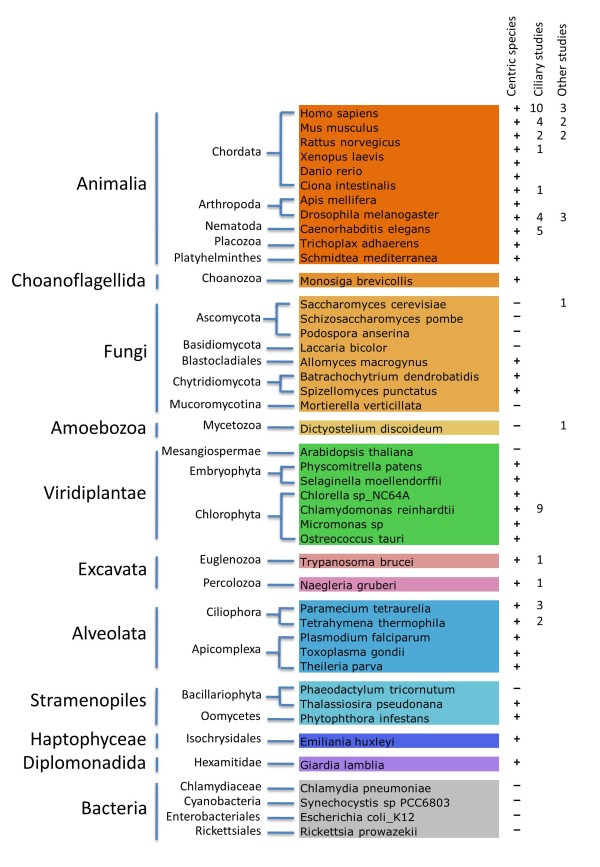
**The species whose whole proteome has been included into Cildb V3.0 are gathered by taxonomy groups, with indication whether they are centric or not and of the number of high throughput studies, ciliary or not, performed in the species.** The choice of species to include into Cildb was 1) species in which high throughput ciliary studies have been performed, 2) species routinely used as models in ciliary studies in general, and 3) centric and acentric species, because the presence/absence of certain proteins may be relevant for the conservation of ciliary proteins through evolution. The case of the Bug22/GTL3/C16orf80 protein, composed of a domain called DUF667, essential for ciliary motility [6], was carefully examined for the choice of fungi to add in Cildb for comparative genomics. Bug22 is a protein highly conserved in all centric species, be they metazoans, protozoa, plants or fungi and curiously also highly conserved in the acentric land plants, but absent from the genomes of higher fungi already sequenced at the time of the publication, i.e. acentric ascomycetes [6]. Owing to constant new genome sequencing, novel fungal whole proteomes appeared and the occurrence of Bug22 was different from what was thought earlier. It is still undetectable in ascomycetes, but is found conserved in the acentric *Mortierella verticillata* (accession MVEG_01915), and a more divergent Bug22 with recognizable DUF667 domain is found in several basidiomycetes represented in Cildb by *Laccaria bicolor* (accession 598201). This property was one of the reasons to include those two fungi proteomes into Cildb V3.0. This also emphasizes that constant arrival of new knowledge as new genomes are sequenced can put into questions former assumptions such as the absence of particular proteins in some species, here Bug22 in fungi.

Since the last version of Cildb, new high throughput ciliary studies have appeared and more model organisms have been used for ciliary studies. Thus, we remodeled Cildb to include the proteomes of altogether 44 species, among which are 41 eukaryotes and 3 bacteria (http://cildb.cgm.cnrs-gif.fr/v3/cgi/genome_versions; Figure 1) and 66 studies, among which 55 directly concern cilia, and 11 other, related studies (http://cildb.cgm.cnrs-gif.fr/v3/cgi/ciliary_studies; Table 1). BLAST server and human GBrowse facilities are maintained in the new version. In addition, a Motif Search tool has been implemented in order to search proteomes with a sequence motif using the patmatdb program from the EMBOSS package (http://bioweb2.pasteur.fr/docs/EMBOSS/patmatdb.html), based on the format of pattern used in the PROSITE database (http://prosite.expasy.org/prosuser.html). For example, an amino acid motif such as MKK[KP]K, in which either K or P can stand at the fourth position, can be queried in the proteome of any species of Cildb.

**Table 1:** High throughput studies compiled in Cildb V3.0

**Reference for the study**	**Method**	**Species**	**Ciliary analysis**
Andersen et al., 2003 [3]	Centriole proteome	*Homo sapiens*	yes
Arnaiz et al., 2009 [1]	Cilium proteome	*Paramecium tetraurelia*	yes
Arnaiz et al., 2010 [7]	Expression during ciliogenesis	*Paramecium tetraurelia*	yes
Avidor-Reiss et al., 2004 [8]	Comparative genomics	*Drosophila melanogaster*	yes
Baker et al., 2008a [9]	Spermatozoa proteome	*Mus musculus*	no
Baker et al., 2008b [10]	Spermatozoa proteome	*Rattus norvegicus*	no
Bechstedt et al., 2010 [11]	Expression in tissues containing sensory cilia	*Drosophila melanogaster*	yes
Blacque et al., 2005 [12]	Differential expression between ciliated and non ciliated cells	*Caenorhabditis elegans*	yes
Blacque et al., 2005 [12]	Genomic screening for X-boxes in promoters	*Caenorhabditis elegans*	yes
Boesger et al., 2009 [13]	Flagellum phosphoproteome	*Chlamydomonas reinhardtii*	yes
Broadhead et al., 2006 [14]	Flagellum proteome	*Trypanosoma brucei*	yes
Cachero et al., 2011 [15]	Expression in early development of future neural cells	*Drosophila melanogaster*	no
Cao et al., 2006 [16]	Sperm flagellar axonemes proteome	*Mus musculus*	yes
Chen et al., 2006 [17]	Expression in daf-19 mutant	*Caenorhabditis elegans*	yes
Datta et al., 2011 [18]	Gene expression with HIPPI expression modulation	*Homo sapiens*	no
Dorus et al., 2006 [19]	Spermatozoa proteome	*Drosophila melanogaster*	no
Efimenko et al., 2005 [20]	Genomic screening for X-boxes in promoters	*Caenorhabditis elegans*	yes
Fritz-Laylin and Cande, 2010 [21]	Flagellum proteome	*Naegleria gruberi*	yes
Geremek et al., 2011 [22]	Expression in primary ciliary dyskinesia patients	*Homo sapiens*	yes
Geremek et al., 2014 [23]	Expression in primary ciliary dyskinesia patients	*Homo sapiens*	yes
Guo et al., 2010 [24]	Proteomics associated with spermiogenesis	*Mus musculus*	no
Hodges et al., 2011 [25]	Comparative genomics	*Chlamydomonas reinhardtii*	yes
Hoh et al., 2012 [26]	Expression in multiciliated cells from trachea	*Mus musculus*	yes
Huang et al., 2008 [27]	Proteomics associated with spermiogenesis	*Mus musculus*	no
Hughes et al., 2008 [28]	Proteome of Microtubule-Associated Proteins	*Drosophila melanogaster*	no
Ishikawa et al., 2012 [29]	Primary cilium proteome	*Mus musculus*	yes
Ivliev et al., 2012 [30]	Expression profile in different tissues	*Homo sapiens*	yes
Jakobsen et al., 2011 [31]	Centrosome proteomics	*Homo sapiens*	yes
Keller et al., 2005 [32]	Expression during ciliogenesis	*Chlamydomonas reinhardtii*	yes
Keller et al., 2005 [32]	Basal body proteome	*Chlamydomonas reinhardtii*	yes
Kilburn et al., 2007 [33]	Basal body proteome	*Tetrahymena thermophila*	yes
Kim et al., 2010 [34]	Ciliogenesis modulation	*Homo sapiens*	yes
Kubo et al., 2008 [35]	Expression in ciliated tissues	*Homo sapiens*	yes
Laurençon et al., 2007 [36]	Genomic screening for X-boxes in promoters	*Drosophila melanogaster*	yes
Lauwaet et al., 2011 [37]	Homology search for basal body proteins	*Giardia lamblia*	yes
Lauwaet et al., 2011 [37]	Basal body proteome	*Giardia lamblia*	yes
Li et al., 2004 [38]	Comparative genomics	*Chlamydomonas reinhardtii*	yes
Liu et al., 2007 [39]	Cilium proteome	*Mus musculus*	yes
Martínez-Heredia et al., 2006 [40]	Spermatozoa proteome	*Homo sapiens*	no
Mayer et al., 2008 [41]	Cilium proteome	*Rattus norvegicus*	yes
Mayer et al., 2009 [42]	Cilium proteome	*Rattus norvegicus*	yes
McClintock et al., 2008 [43]	Expression in ciliated tissues	*Mus musculus*	yes
Merchant et al., 2007 [44]	Comparative genomics	*Chlamydomonas reinhardtii*	yes
Müller et al., 2010 [45]	Centrosome proteome	*Drosophila melanogaster*	yes
Nakachi et al., 2011 [46]	Sperm tail proteome	*Ciona intestinalis*	yes
Nogales-Cadenas et al., 2009 [47]	Centrosome human curation	*Homo sapiens*	yes
Ostrowski et al., 2002 [2]	Cilium proteome	*Homo sapiens*	yes
Pazour et al., 2005 [5]	Expression during ciliogenesis	*Chlamydomonas reinhardtii*	yes
Pazour et al., 2005 [5]	Flagellum proteome	*Chlamydomonas reinhardtii*	yes
Phirke et al., 2011 [48]	Down and upregulated genes in daf-19 mutant	*Caenorhabditis elegans*	yes
Reinders et al., 2006 [49]	Nuclear-associated body proteome	*Dictyostelium discoideum*	no
Ross et al., 2007 [50]	Expression during ciliogenesis	*Homo sapiens*	yes
Sakamoto et al., 2008 [51]	Proteome of Microtubule-Associated Proteins	*Rattus norvegicus*	no
Sauer et al., 2005 [52]	Mitotic spindle proteome	*Homo sapiens*	no
Smith et al., 2005 [53]	Cilium proteome	*Tetrahymena thermophila*	yes
Stolc et al., 2005 [54]	Expression during ciliogenesis	*Chlamydomonas reinhardtii*	yes
Stubbs et al., 2008 [55]	Expression Under FoxJ1 silencing	*Xenopus laevis*	yes
Wigge et al., 1998 [56]	Spindle pole body proteome	*Saccharomyces cerevisiae*	no
Yano et al., 2013 [57]	Ciliary membrane proteome	*Paramecium tetraurelia*	yes

The high throughput studies present in Cildb V3.0 are summarized in the table with indication in the second column whether it is a proteomic, gene expression, or genomic study. The species in which the studies have been performed are specified in the third column. In the fourth column is the fact whether a given study is ciliary (concerns cilia, flagella, basal bodies, centrioles, centrosomes or spindle pole bodies) or not. The table is ordered alphabetically by first author of publication of the studies present in Cildb V3.0.

#### Species implemented in Cildb V3.0

Cildb V3.0 contains now whole proteomes of 41 eukaryotes among which 32 are centric species. Fifteen of these species were used for the 66 high throughput studies of Cildb. The 17 other species are good models for ciliary experiments although no high throughput study has been published as of yet. Nine eukaryotic acentric species which lack cilia and centrioles were also taken because they represent ‘negative controls’ in comparative genomics experiments: two species for which two analyses on spindle pole proteomes are available and seven species without high throughput relevant studies.

Since orthology relationships are a major tool in Cildb, we corrected an inconsistency in the proteome composition in various species. Indeed, species present in Cildb are not homogeneous in their whole proteome, some of them including organelle proteomes (mitochondria, chloroplasts), others not. Organelle proteomes represent a minor part of all the proteins, but since some organellar proteins can be encoded either by nuclear genes or by the organelle, according to the species, this may influence the orthology calculation in some cases. This issue has been fixed in Cildb V3.0. In addition, to study the origin of organellar proteins, we added the whole proteomes of three bacteria because they are closest to those of mitochondria (*Rickettsia prowazekii*) and chloroplasts (*Synechocystis sp PCC6803*, *Chlamydia pneumoniae*).

Since the original publication of Cildb [1], the whole proteomes of 26 novel eukaryotic species have been introduced into Cildb. A notable proportion of fungi, eight fungal whole proteomes, are incorporated in Cildb mainly because fungi represent a phylum at a hinge position in the evolution of centric and acentric species.

#### Studies in Cildb V3.0

The 66 studies incorporated in Cildb V3.0 mainly consist in high throughput proteomics, differential expression, and comparative genomics studies. 53 of these studies approach ciliary and centriolar/basal body components, structure, function or biogenesis. We also integrated 13 studies concerning related topics, such as microtubule-associated proteins, spindle proteins, spindle pole bodies, nuclear-associated bodies, whole sperm proteome, and others. Compared to Cildb V1.0, 45 novel studies have been introduced in Cildb.

High throughput studies concerning cilia appear monthly in the literature, but computation in Cildb needs full recalculation of the database, so that it cannot be updated each time. However, if the output of a study not present in Cildb has to be compared to a study already present, this can be performed using the keyword box in the general properties filter by querying a list of gene or protein IDs bordered by ‘%’, one per line. The limitation is that the query is slow, since this is not the main task designed for BioMart queries.

### Simplified interface and structure for Cildb V3.0

**Figure 2:**
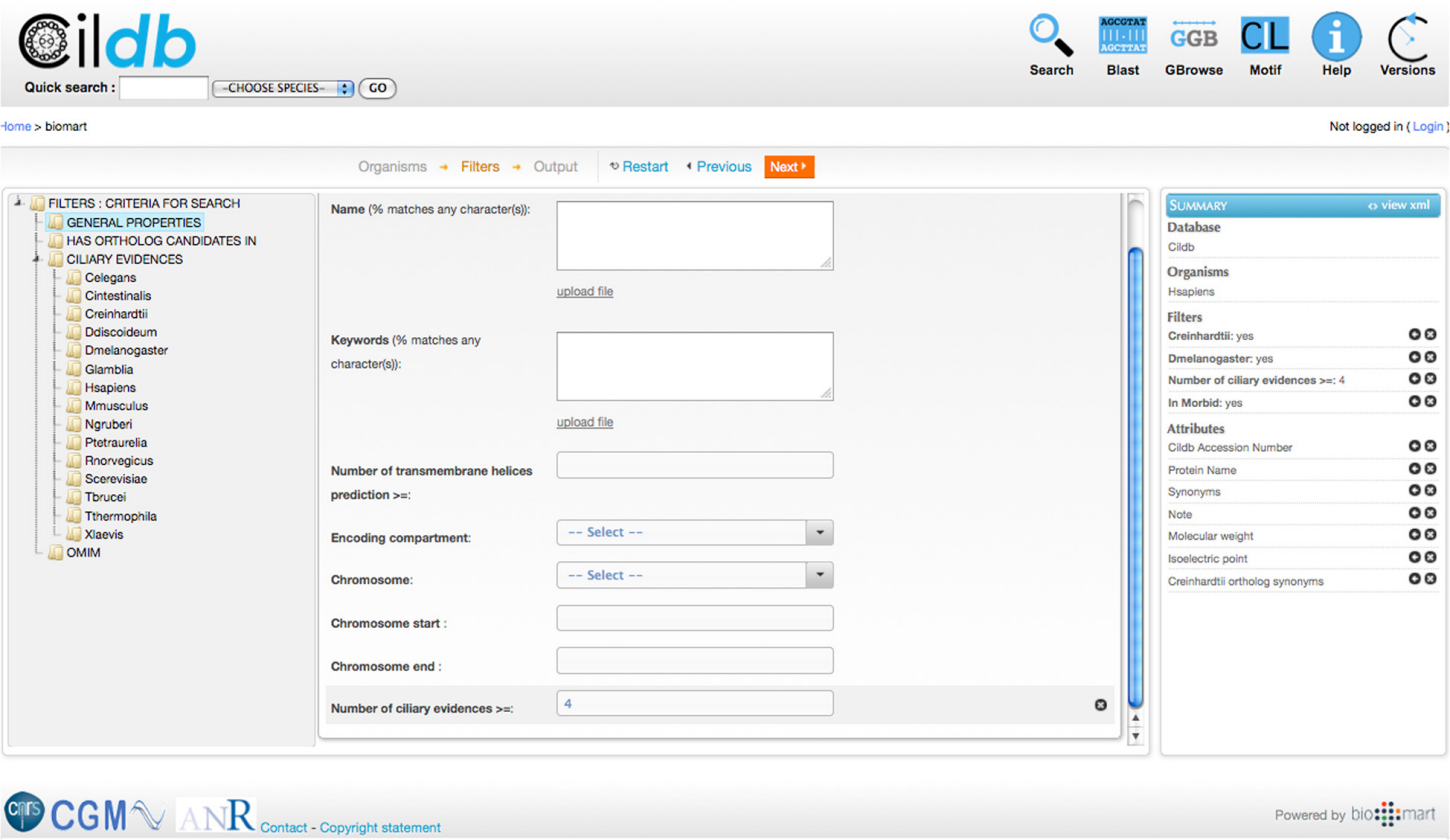
**An advanced search on Cildb V3.0 is started by clicking on the ‘Search’ button on the top row on the right.** Then, it is necessary to choose the species in which the proteome has to be searched for. The filter window then appears to adjust the filters in the left panel (no filter means that the full proteome will be retrieved). Similarly, the output window allows displaying particular properties (attributes) in columns for each filtered protein. A summary on the right reminds the user of all the filters and attributes currently used. This also allows direct modification of the orders of the columns in the output by moving the attributes up and down in the list. The last operation of the process is to show the results. The results are given by pages of 20 items with a maximum of 1000 items. To see all results, they have to be downloaded as a file. At any time, if the result output seems incomplete or inappropriate, the filters and attributes can be modified by using the ‘Back’ button (edit results) to refine the search and show the results again. The quick search allows a rapid search by keywords. The result can be processed the same way as the one described above, with the possibility to add attributes by ‘Edit results’ and to download the file. Note the direct access to BLAST, Human genome Gbrowse, Motif search, Help and access to older Versions of Cildb on the top row buttons to the right.

For users trained with previous versions of Cildb, the most prominent change is the new interface. Indeed, it takes advantage of the novel environment provided by BioMart Version 9 [58] (Figure 2). In consequence, making an advanced search becomes much more intuitive than earlier, even for non-trained users, who can easily enter the functionalities of the database.

The simplification of the interface is accompanied by a simplification of the structure of the database. First of all, the orthology calculation has been exclusively centered on Inparanoid [4]. Formerly, users could choose between Inparanoid and Inparanoid plus ‘in house’ filtered blast hits. The most recent version of Inparanoid appears efficient enough to prevent the output of too many false negatives that occurred with the previous versions, so that the addition of ‘in house’ filtered blast hits was no more necessary, as detailed in the next section and in the legend of Table 2. We also simplified the way to filter ciliary studies and removed less useful other searches (operator ‘OR’, customized searches). However, the functions removed in the query menu compared to previous Cildb versions can be applied by another process that consists of downloading data as tables with relevant attributes and sorting these tables thereafter using a spreadsheet software.

**Table 2:** Evolutionary conservation of centrosomal proteins viewed through Cildb V3.0

	**Protein ID**	**Synonyms**	**Mus**	**Rattus**	**Danio**	**Apis**	**Drosophila**	**Class**
1	ENSP00000380378	PAFAH1B1,LIS1,LIS2,MDCR	yes	yes	yes	yes	yes	1 (yyyyy)
2	ENSP00000364691	CROCC,ROLT,ROLT,rootletin	yes	yes	yes	yes	yes	1 (yyyyy)
3	ENSP00000309591	PRKACA,PKACA,PKACa	yes	yes	yes	yes	yes	1 (yyyyy)
4	ENSP00000263710	CLASP1,MAST1	yes	yes	yes	yes	yes	1 (yyyyy)
5	ENSP00000263811	DYNC1I2,DNCI2,IC2	yes	yes	yes	yes	yes	1 (yyyyy)
6	ENSP00000216911	AURKA,AIK,ARK1,AURA,AURORA2	yes	yes	yes	yes	yes	1 (yyyyy)
7	ENSP00000364721	MAPRE1,EB1,EB1	yes	yes	yes	yes	yes	1 (yyyyy)
8	ENSP00000265563	PRKAR2A,PKR2,PRKAR2	yes	yes	yes	yes	yes	1 (yyyyy)
9	ENSP00000355966	NEK2,HsPK21,NEK2A,NLK1	yes	yes	yes	yes	yes	1 (yyyyy)
10	ENSP00000261965	TUBGCP3,GCP3,SPBC98	yes	yes	yes	yes	yes	1 (yyyyy)
11	ENSP00000252936	TUBGCP2,GCP2,Grip103,h103p	yes	yes	yes	yes	yes	1 (yyyyy)
12	ENSP00000251413	TUBG1,CDCBM4,GCP-1	yes	yes	yes	yes	yes	1 (yyyyy)
13	ENSP00000456648	TUBGCP4,76P,GCP-4,GCP4	yes	yes	yes	yes	yes	1 (yyyyy)
14	ENSP00000323302	POC1B,PIX1,TUWD12,WDR51B	yes	yes	yes	yes	yes	1 (yyyyy)
15	ENSP00000324464	CSNK1D,ASPS,CKIdelta,FASPS2,HCKID	yes	yes	yes	yes	yes	1 (yyyyy)
16	ENSP00000270861	PLK4,SAK,STK18,Sak	yes	yes	yes	yes	yes	1 (yyyyy)
17	ENSP00000356785	NME7,MN23H7,NDK7	yes	yes	yes	yes	yes	1 (yyyyy)
18	ENSP00000273130	DYNC1LI1,DNCLI1,LIC1	yes	yes	yes	yes	yes	1 (yyyyy)
19	ENSP00000359300	CETN2,CALT,CEN2	yes	yes	yes	yes	yes	1 (yyyyy)
20	ENSP00000287380	TBC1D31,Gm85,WDR67	yes	yes	yes	yes	yes	1 (yyyyy)
21	ENSP00000287482	SASS6,SAS-6,SAS6	yes	yes	yes	yes	yes	1 (yyyyy)
22	ENSP00000300093	PLK1,PLK,STPK13	yes	yes	yes	yes	yes	1 (yyyyy)
23	ENSP00000257287	CEP135,CEP4,MCPH8	yes	yes	yes	yes	yes	1 (yyyyy)
24	ENSP00000439376	DCTN2,DCTN50,DYNAMITIN,RBP50	yes	yes	yes	yes	yes	1 (yyyyy)
25	ENSP00000395302	CKAP5,ch-TOG,CHTOG,MSPS	yes	yes	yes	yes	yes	1 (yyyyy)
26	ENSP00000342510	CEP97,LRRIQ2	yes	yes	yes	yes	yes	1 (yyyyy)
27	ENSP00000348965	DYNC1H1,DHC1,DHC1a	yes	yes	yes	yes	yes	1 (yyyyy)
28	ENSP00000469720	CETN2,CALT,CEN2	yes	yes	yes	yes	yes	1 (yyyyy)
29	ENSP00000317156	CEP192,PPP1R62	yes	yes	yes	yes	no	2 (yyyyn)
30	ENSP00000270708	WRAP73,WDR8	yes	yes	yes	yes	no	2 (yyyyn)
31	ENSP00000248846	TUBGCP6,GCP-6,GCP6,MCCRP,MCPHCR	yes	yes	yes	yes	no	2 (yyyyn)
32	ENSP00000393583	AZI1,AZ1,Cep131,ZA1	yes	yes	yes	yes	no	2 (yyyyn)
33	ENSP00000283645	TUBGCP5,GCP5	yes	yes	yes	yes	**no**	2 (yyyyn)
34	ENSP00000303058	CEP120,CCDC100	yes	yes	yes	yes	no	2 (yyyyn)
35	ENSP00000313752	SSNA1,N14,NA-14	yes	yes	yes	yes	no	2 (yyyyn)
36	ENSP00000355812	FGFR1OP,FOP	yes	yes	yes	yes	no	2 (yyyyn)
37	ENSP00000343818	CDK5RAP2,C48,Cep215,MCPH3	yes	yes	yes	**no**	yes	3 (yyyny)
38	ENSP00000344314	OFD1,CXorf5,JBTS10,RP23	yes	yes	yes	no	no	4 (yyynn)
39	ENSP00000317144	PIBF1,C13orf24,CEP90	yes	yes	yes	no	no	4 (yyynn)
40	ENSP00000204726	GOLGA3,GCP170,MEA-2,golgin-160	yes	yes	yes	no	no	4 (yyynn)
41	ENSP00000206474	HAUS4,C14orf94	yes	yes	yes	no	no	4 (yyynn)
42	ENSP00000281129	CEP128,C14orf145,C14orf61,LEDP/132	yes	yes	yes	no	no	4 (yyynn)
43	ENSP00000262127	CEP76,C18orf9,HsT1705	yes	yes	yes	no	no	4 (yyynn)
44	ENSP00000370803	CCP110,Cep110,CP110	yes	yes	yes	no	no	4 (yyynn)
45	ENSP00000263284	CCDC61	yes	yes	yes	no	no	4 (yyynn)
46	ENSP00000223208	CEP41,JBTS15,TSGA14	yes	yes	yes	no	no	4 (yyynn)
47	ENSP00000303769	AKNA	yes	yes	yes	no	no	4 (yyynn)
48	ENSP00000302537	MDM1	yes	yes	yes	no	no	4 (yyynn)
49	ENSP00000264935	CEP72,FLJ10565	yes	yes	yes	no	no	4 (yyynn)
50	ENSP00000419231	CEP70,BITE	yes	yes	yes	no	no	4 (yyynn)
51	ENSP00000306105	CEP89,CCDC123	yes	yes	yes	no	no	4 (yyynn)
52	ENSP00000380661	CEP250,C-NAP1,CEP2,CNAP1	yes	yes	yes	no	no	4 (yyynn)
53	ENSP00000356579	CEP350,CAP350,GM133	yes	yes	yes	no	no	4 (yyynn)
54	ENSP00000260372	HAUS2,C15orf25,CEP27,HsT17025	yes	yes	yes	no	no	4 (yyynn)
55	ENSP00000360540	CEP55,C10orf3,CT111,URCC6	yes	yes	yes	no	no	4 (yyynn)
56	ENSP00000355500	CEP170,FAM68A,KAB	yes	yes	yes	no	no	4 (yyynn)
57	ENSP00000369871	HAUS6,Dgt6,FAM29A	yes	yes	yes	no	no	4 (yyynn)
58	ENSP00000371308	CENPJ,BM032,CENP-J,CPAP,LAP,LIP1,MCPH6,Sas-4	yes	yes	yes	no	no	4 (yyynn)
59	ENSP00000282058	HAUS1,CCDC5,HEI-C,HEIC	yes	yes	yes	no	no	4 (yyynn)
60	ENSP00000283122	CETN3,CDC31,CEN3	yes	yes	yes	no	no	4 (yyynn)
61	ENSP00000352572	PCNT,KEN,MOPD2,PCN,PCNT2,PCNTB	yes	yes	yes	no	no	4 (yyynn)
62	ENSP00000295872	SPICE1,CCDC52,SPICE	yes	yes	yes	no	no	4 (yyynn)
63	ENSP00000317902	CEP57,MVA2,PIG8,TSP57	yes	yes	yes	no	no	4 (yyynn)
64	ENSP00000426129	CEP63	yes	yes	yes	no	no	4 (yyynn)
65	ENSP00000308021	CEP290,BBS14,JBTS5,LCA10,MKS4,NPHP6,POC3,rd16,SLSN6	yes	yes	yes	no	no	4 (yyynn)
66	ENSP00000439056	HAUS5,dgt5	yes	yes	yes	no	no	4 (yyynn)
67	ENSP00000462740	CEP41,JBTS15,TSGA14	yes	yes	yes	no	no	4 (yyynn)
68	ENSP00000265717	PRKAR2B,PRKAR2,RII-BETA	yes	yes	no	yes	yes	5 (yynyy)
69	ENSP00000345892	NDE1,HOM-TES-87,LIS4,NDE,NUDE	yes	yes	no	yes	yes	5 (yynyy)
70	ENSP00000358921	ACTR1A,ARP1,CTRN1	yes	yes	no	yes	yes	5 (yynyy)
71	ENSP00000447907	DYNLL1,DLC1,DLC8,DNCL1,DNCLC1,hdlc1,LC8	yes	yes	no	no	no	6 (yynnn)
72	ENSP00000278935	CEP164,NPHP15	yes	yes	no	no	no	6 (yynnn)
73	ENSP00000264448	ALMS1,ALSS	yes	yes	no	no	no	6 (yynnn)
74	ENSP00000316681	KIAA1731	yes	yes	no	no	no	6 (yynnn)
75	ENSP00000456335	CNTROB,LIP8,PP1221	yes	yes	no	no	no	6 (yynnn)
76	ENSP00000348573	AKAP9,AKAP350,AKAP450,CG-NAP,HYPERION,LQT11	yes	no	no	no	no	7 (ynnnn)
77	ENSP00000384844	DCTN1,DAP-150,P135	**no**	**no**	yes	yes	yes	8 (nnyyy)

This table presents the list of 77 human proteins obtained from a BioMart search described in the text. The output gives a total of 133 proteins encoded by 77 genes, due to the presence of splice variants. For clarity, only one protein ID per gene has been presented in the table, after verification that all the splice variants of each gene displays the same orthology relationships with the species presented here. This table illustrates evolutionary conservation where a “yes” indicates that the human protein has an Inparalog in Cildb and a “no” that no Inparanoid orthology was found. The column ‘class’ serves to order the output genes in the table (from 5× ‘yes’ at the top to much fewer ‘yes’ at the bottom, along criteria of certain species being closer to each other than others, whereby the order from left to right goes human-mouse-rat (mammals), then fish (vertebrate), then bee and fly (insects). All instances of lacking orthology (“no”) were individually verified by BLAST searches using the Cildb BLAST. The BLAST results were consistent with the absence of orthologs in the species, and only three exceptions contradict the Inparanoid results, highlighted as bold characters in the table.

1- Human Azi1 (ENSP00000393583) has no inparalog in *Drosophila* although an ortholog called *dilatory* exists. BLAST search on the *Drosophila* genome indeed light up *dilatory*, with a score very close to the one found for the *Apis* inparalogs by BLAST. The difference between these different outputs may result from the value of default thresholds taken by the Inparanoid program and the different lengths of the proteins.

2- Human cdk5rap2 (ENSP00000343818) has no Inparalog in* Apis*, although homologs are found by BLAST. Inparanoid relationships of the top three Apis proteins in the list (XP_006563202.1, XP_006563201.1, XP_392107.3) appear to be Inparalogs of *Drosophila* centrosomin (cnn, cdk5rap2) for which 8 of 12 splice variant proteins display human Inparalogs. However, no direct Inparanoid relationships exist between the Apis proteins and any human protein.

3- Human dynactin/dctn1 (ENSP00000384844) has surprisingly no Inparalogs in mouse and rat whereas some are found in fish, bee and fly. However, mouse and rat homologs are easily found by BLAST search. After careful examination, it appears that the only ENSP00000384844 dynactin protein found common to the three human centrosomal studies, is one of the splice variants excluded from Inparalog groups. Indeed, the 16 splice variants for the human dynactin gene *ENSG00000204843* and the seven splice variants for its mouse counterpart *ENSMUSG00000031865* are related by Inparanoid orthology through three groups, *hsap_mmus.17187* (one human and one mouse gene), *hsap_mmus.1073* (four human and one mouse gene) and *hsap_mmus.977* (one human and two mouse genes). The remaining ten human protein variants (among which is ENSP00000384844) and three mouse protein variants encoded by these genes are not included in the orthology groups, probably because their exon composition was too different from the other protein variants.

These three examples represent the limits of Inparanoid orthology prediction, highlighting the fact that reciprocal BLAST searches cannot be avoided, and thus represent an important complementary approach, for the analysis of individual proteins.

The changes brought to Cildb may have unexpected impact and we would be grateful for any feedback by the users. In addition, since genome annotations evolve with time, proteins can be gained or lost in the deduced proteomes from a time to the next. For all these reasons, we kept the former “data freeze” versions of Cildb available through the “Version” menu for comparisons when it is necessary.

### Evolutionary conservation viewed through Cildb, the example of centrosomal proteins

To evaluate the identification of orthologs by Inparanoid, called ‘inparalogs’, we studied centrosomal proteins in more detail, since they are conserved proteins already pretty well known. We wondered whether centrosomal proteins identified in three studies in *Homo sapiens* would reveal the orthologs, when they exist, in other species. We used the following protocol:

click the ‘Search’ button on the bar on the to right

select ‘Hsapiens’ as organism in the scroll-down menu

click ‘Next’ and open ‘Ciliary Evidences’ on the left menu

click ‘Hsapiens’ and select ‘yes’ for the centrosomal studies [3,31] and [47]

click ‘Next’ and display ortholog names, synonyms, etc. for any desired species listed in the left menu. You can select here as an output the stringency for the studies chosen in the queries, if you want to sort the output table thereafter.

click ‘Results’ to visualize the output

modification of the filters and output can be obtained by the back button ‘Edit Results’

when satisfied with the result, click ‘Download data’

We chose to emphasize the orthologs in *Mus musculus*, *Rattus norvegicus*, *Danio rerio*, *Apis mellifera* and *Drosophila melanogaster* in the output to follow the evolutionary conservation, as viewed with Inparanoid. Among the 113 human proteins encoded by 77 genes found as centrosomal by this filter, inparalogs were detected for 76 genes in mouse, 75 in rat, 68 genes in fish, 37 genes in bee and 33 genes in fly (Table 2). A vast majority of these proteins were identified in mammals, as well as in fish, a vertebrate. More negative examples were found in the insects bee and fly. To check whether homologues were indeed absent when no Inparalogs were found, we performed BLAST searches on individual species proteomes using the Cildb BLAST. Except for the two cases discussed in the legend of Table 2, all the absence of Inparalogs corresponds to no or weak BLAST hit detection. In addition, none of the BLAST targets were found in the previous version of Cildb as filtered best hits, a calculation method that we suppress in the present version. Altogether, although reciprocal BLAST searches are always useful to study the occurrence of individual proteins in various species, the orthology calculation via Inparanoid is pretty suitable for batch identification of conserved proteins using Cildb.

## Conclusion

The version V3.0 of Cildb preserves its major original principles of relating orthology to ciliary studies, but, by improving its structure and its interface, makes the database more suitable for advanced searches. Altogether, Cildb V3.0 is a particularly useful tool for unraveling ciliary and ciliopathy networks and will hopefully help in identification of new orphan diseases.

## Competing interests

The authors declare that they have no competing interests.

## Authors’ contributions

OA made bioinformatics calculations and developed, designed the database, JC and FK brought the biological knowledge on ciliary high throughput studies and species relevant to be included in the database, AMT validated the present version of the database concerning orthology of ciliary and centrosomal conserved proteins viewed by Inparanoid, JC, FK and AMT wrote the manuscript. All authors read and approved the final manuscript.
